# Understanding large scale sequencing datasets through changes to protein folding

**DOI:** 10.1093/bfgp/elae007

**Published:** 2024-03-23

**Authors:** David Shorthouse, Harris Lister, Gemma S Freeman, Benjamin A Hall

**Affiliations:** School of Pharmacy, University College London, 29-39 Brunswick Square, London WC1N 1AX, UK; Department of Medical Physics and Biomedical Engineering, Malet Place Engineering Building, University College London, Gower Street, London WC1E 6BT, UK; Department of Medical Physics and Biomedical Engineering, Malet Place Engineering Building, University College London, Gower Street, London WC1E 6BT, UK; Department of Medical Physics and Biomedical Engineering, Malet Place Engineering Building, University College London, Gower Street, London WC1E 6BT, UK

**Keywords:** genomics, mutation, folding, DDG, structure

## Abstract

The expansion of high-quality, low-cost sequencing has created an enormous opportunity to understand how genetic variants alter cellular behaviour in disease. The high diversity of mutations observed has however drawn a spotlight onto the need for predictive modelling of mutational effects on phenotype from variants of uncertain significance. This is particularly important in the clinic due to the potential value in guiding clinical diagnosis and patient treatment. Recent computational modelling has highlighted the importance of mutation induced protein misfolding as a common mechanism for loss of protein or domain function, aided by developments in methods that make large computational screens tractable. Here we review recent applications of this approach to different genes, and how they have enabled and supported subsequent studies. We further discuss developments in the approach and the role for the approach in light of increasingly high throughput experimental approaches.

## INTRODUCTION

DNA sequencing has revolutionized biomedical research. The ability to routinely sequence samples with increasing sensitivity and continually reducing costs has enabled major new insights into diseases and ageing. In turn, this has led to a growth in the routine use of sequencing in the clinic; for example, in the UK, the NHS now routinely sequences at risk prenatal foetuses [1] for mutations in a panel of genes known to cause disorders, and this type of testing is now taking place in several countries [2]. This collection of data is likely to become increasingly widespread as costs continue to decline, and with that become more available in lower-middle income countries. In principle this deluge of data should improve our understanding of disease, as we become able to link variants to specific clinical phenotypes. However, a fundamental issue with genomic variants is in their interpretability. It is estimated that roughly two-thirds of disease causing variants are nucleotide substitutions, causing either a truncation of the protein (e.g. through introducing a termination codon, or a splice site) or a substitution of amino acids in the protein sequence (missense mutations) [3]. Missense mutations can cause either loss or gain of resultant protein activity, or not alter gene function at all. Whilst some missense mutations occur reliably in hotspots, the wide diversity of variants that present in the clinic lead to problems of interpreting variants of uncertain significance (VUS). This is a problem for relatively common diseases, such as the RASopathy Noonan’s syndrome (effecting 1 in 2000 individuals), where almost 60% of prenatally detected mutations in associated genes were found to be VUS [4], but presents an even more serious barrier for diagnosis of rare diseases. Given that the frequency of rare diseases as a cohort is high, effecting 3.5–5.9% of individuals globally [5], interpretation and confirmation of the role of a missense mutation in disease is paramount. This is of particular importance for genes that are not tractable to assay (e.g. many membrane proteins), where the commonly used assay does not reflect all aspects of the disease (e.g. assays for fumarate hydratase function, [6]), or where collection of materials is not possible (e.g. RASopathies, which can present prenatally [7]).

Bioinformatic and genomic approaches offer one route to understanding such variants. FATHMM [8], GenePy [9] and PolyPhen-2 [10] are examples of statistical tools to predict pathogenicity of variants, using machine learning algorithms trained on recorded observations. Whilst these approaches are powerful, they also share common limitations. Whilst they can illustrate statistical correlations between variant sites and types and disease, they do not offer biophysical mechanisms for variant action. This is necessary for understanding the type of perturbation caused by the variant- an ‘edgetic’ modification would influence a specific function or protein interaction, whilst ‘global’ modifications would alter overall levels of gene activity on all downstream elements [11]. The type of mutation is associated with specific clinical phenotypes or cellular behaviours, and understanding this is therefore necessary for predicting clinical outcomes. Understanding specific mechanisms can also provide routes to drug development. For example, mutations to the gene GLA that cause protein misfolding can be successfully treated with the chaperone migalastat [12], but mutations that alter enzyme activity in other ways do not respond. Finally, to date, sequencing data have predominantly been collected in advanced countries with widespread access to healthcare. This creates fundamental problems of bias in the underlying data, limiting the transferability of statistical approaches [13].

**Figure 1:**
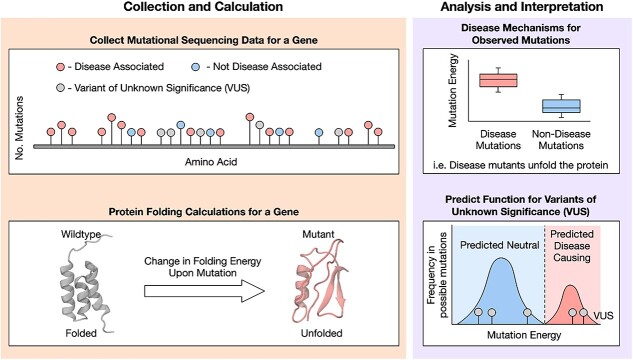
Schematic demonstrating the role of protein folding calculations in interpreting results from genetic sequencing data.

Molecular modelling approaches can address these issues. Based on the 3D atomic structure of the protein, they can be used to link observed variants to specific parts of the structure–function relationship. Most approaches in the field focus on the study of single or small groups of variants, modelled in highly complex systems and performed on high performance computing facilities. Whilst these can offer insights into single variants, computational costs are extremely high, making translation to large numbers of variants difficult, and the large amount of time needed to perform calculations limits the potential for integration into the clinical diagnosis pipeline. Despite this, molecular modelling enables bridging between mutational data and protein function, and several techniques have demonstrated the ability to achieve this now (e.g. protein insertion into membranes [14], single helix simulations [15], multi-helix simulations [16], protein binding [17]). The calculation of changes to protein folding energies upon amino acid changes, the Gibbs free energy (ΔΔG), is now well established as a tool for predicting the impact of large sets of mutations on the folding ability of a protein (Figure 1). Prevention of canonical protein folding is a fundamental mechanism for altering protein activity. Incorrect folding is estimated to be responsible for loss of activity in two-thirds of variants across multiple long standing studies [18–21], and is responsible for the majority of pathogenic missense mutations observed in humans [22]. A wide range of tools are now available for calculating folding energies of protein structures, with tools such as Rosetta [23] and FoldX [24] dominating the landscape (see approaches summarized in Table 1).

**Table 1:** Methods for computing the energy change of folding on mutation

**Method**	**Computational Cost for Saturation Screen**	**Comments**
Forcefield-based methods (e.g. Foldx)	Low – 10-100 s of CPU hours per structure	High efficiency, easy to parallelize, based on a set of energy parameters (forcefield), no incorporation of dynamics
Ensemble-based methods (e.g. Rosetta FlexDDG)	Medium – 1000-10 000 s of CPU hours per structure	Medium efficiency, possible to parallelize, collects ensembles of structures from small-scale dynamics calculations to generate energies
Dynamics-based methods (e.g. Alchemical methods)	High – >10 000 s of CPU hours per structure	Poor efficiency for high-throughput, requires extensive CPU time for each mutation, can be parallelized, mutates a wildtype protein over a small molecular dynamics simulation

In this review, we draw together multiple examples of how this approach enables predictive insights in human disease. This is not an exhaustive review of papers deploying these methods, but for single diseases we focus on papers where specific insights or data derived from protein folding calculations have been reused by others to make discoveries or aid clinical interpretations. We further discuss studies that examine the aggregate properties of multiple genes, either to identify global trends induced by mutations, or to integrate folding data into more complex models of gene interaction. Finally, we discuss upcoming developments that are likely to transform how the method is applied.

## UNDERSTANDING DISEASE CAUSING VARIANTS THROUGH MISFOLDING CALCULATIONS

Hereditary diffuse gastric cancer is an inherited condition that can lead to sporadic diffuse stomach and lobular breast cancers, and is driven by loss of function mutations to the *CDH1* gene encoding E-cadherin. Following previous *in vitro* work from the same group, and motivated in part by potential artefacts induced by the assays used there, Simões-Correia *et al.* used FoldX to calculate the impact of mutations on E-cadherin stability [25]. Based on the limited structural data available at the time, a homology model of the E-cadherin protein was built from the SWISS-MODEL repository [26]. The protein prodomain is cleaved from the protein during normal maturation, and the authors found that mutations observed in the prodomain are not associated with destabilization, consistent with the region not having a role in activity post maturation. A total of 70% of mutations to the extracellular domain were found to be predicted as pathogenic as determined by high mutational ΔΔG estimates and a simple cutoff threshold, and that destabilization of the protein was associated with a younger age of onset of the disease. Based on these calculations three previously uncharacterized variants, two predicted neutral and one predicted destabilizing, were validated *in vitro*, demonstrating that the predictions were correct. The authors further identified a mutation at the same site of a known deleterious variant that was expected to be stable, and confirmed experimentally that this alternative missense mutation was indeed tolerated. This work was followed up by Figueiredo, who adapted similar experimental and theoretical approaches to study one missense variant [27]. However, arguably the most substantial impact of this notable work is the impact on assessment and curation guidelines for mutations of *CDH1* ([28], reviewed in [29]). These explicitly used the evidence taken from this work to alter the rules for clinical classification of *CDH1* variants based on predictions from protein folding calculations. This highlights the potential value of computational analysis such as this in assessing VUS when taken alongside other data.

Retinitis pigmentosa (RP) is one of the most common retinopathies, causing progressive loss of peripheral and night vision, which can, in turn, lead to loss of central vision [30]. This is frequently caused by mutations to rhodopsin. Rakoczy *et al.* studied 103 known RP causing mutations in rhodopsin with a mixture of structural bioinformatics tools, including misfolding calculations [31]. A total of 62 of the variants were found to cause protein misfolding, whilst others could be understood in terms of known protein biology or membrane insertion. Notably they observed a clear correlation between calculated ΔΔG values and both vision loss onset and average age of night blindness, suggesting that the severity of the disorder could be understood through the relative destabilization of the protein fold. This work led to several other studies, including experimental exploration of small groups of mutations [32], and high throughput assays that were directly compared with data, challenging some of the conclusions drawn [33]. Perhaps some of the most impactful work that arises from this study however is the insight that treating the misfolding directly might reverse some or all of the symptoms. The use of small chaperone proteins as a therapy was computationally explored in [34], and the wider set of treatments for RP (including chaperone therapy) reviewed in [35].

Fumarate hydratase is an oncoenzyme, whose loss is associated with hereditary leiomyomatosis and renal cell cancer (HLRCC) [36]. This is a late-onset disease, for whom improved mutant classification could support clinical diagnosis of VUS, but in principle routine sequencing could identify individuals at higher risk. Shorthouse *et al.* [21] used misfolding alongside other measures of biophysical properties in the computational equivalent of a multiplexed assay for variant effects (MAVE) to determine the protein structural features that drove disease based on publicly available data from the fumarate hydratase database [37]. This classifier was able to identify three structural features that correctly predicted the impact of mutations- misfolding (accounting for 2/3 of deleterious mutations), modifications proximal to the active site and substitutions at hinges in the protein. Notably, the importance of dynamics had not been observed previously and its analysis was enabled by use of elastic network modelling, verified by molecular dynamics. Furthermore, this approach ruled out possible mechanisms speculated in the literature of the involvement of an allosteric ‘B’ site in this disease [38]. The model was verified against publicly available metabolomics data, and classification and data were made available with publication. This has enabled the reuse of the data in clinical case reports, where novel mutations were assessed and classified using the approach [39]. A more complicated problem for understanding VUS is the known issues in clinical assays used to assess enzyme activity in the clinic, which identify fumarate hydratase deficiency in individuals who do not develop renal cancer [6]. The same computational approach applied to small sets of variants reveals that variants that are identified in the assay but do not lead to cancer have lower misfolding energies than disease causing variants and are distant from the enzyme binding site [40]. This finding is consistent with the variants being sufficiently disruptive to trigger a response in the assay but insufficient to cause more serious disease.

Studies of the COVID spike protein have made extensive use of folding calculations to understand the patterns of evolving pathogenicity. Learning how the spike changes is important both for understanding how COVID adapts to human hosts, but also for its role in immune evasion. One pre-alpha study by Laha *et al.* reported alignments of whole genome sequencing, identifying frequently mutated regions and co-occurrence of mutation pairs between different proteins [41]. Using structures available at the time, models of mutations were constructed using a combination of SWISS-MODEL [26] to model missing loops, and FoldX to introduce point mutations. The impact of mutations were assessed using both FoldX overall energy estimates, alongside empirical approaches that estimated the energy of substitution through summing the impact of specific contacts made and broken and portioning energies. This study made several important contributions that aided the interpretation of later datasets. The identification of the recurrent mutation D614G as a stabilizing substitution supported later work on its impact on viral infectivity [42], and the underlying mechanism of selection was further extended to consider the impact on binding affinity [43]. This observation was used to support several later sequencing studies performed on different populations, supporting the interpretation of novel data as it arose [44–46].

Teng *et al.* followed this work in early 2021 with a detailed theoretical study expanding the approach explicitly to computational saturation mutagenesis applied to the spike protein [47]. Here the researchers used FoldX again to calculate the change in the free energy of folding and the change in binding affinity to the ACE2 receptor, and compared them with predictions of impact from the machine learning based tool SNAP [48], verifying 20 notable mutations against other prediction tools. They found clear correlation between folding energies and SNAP scores, and a bias towards stabilization of the spike protein amongst the observed mutations. This was a powerful resource for several new studies to confirm findings. It supported validation of specific recurrent observations such as the L452R substitution, predicted to be both stabilizing and enhancing of binding to ACE2 [49], and high throughput experimental assays using yeast expression systems [50]. As with Laha *et al.*, the data were important in interpreting sequencing data taken from different subpopulations [51–53], and the approach reapplied to analysis of Middle East respiratory syndrome (MERS) evolution [54]. This work also empowered a new set of analyses, where phylogenies and clades were studied explicitly using insights from the change of binding and folding [55, 56].

The impact of misfolding on viral evolution explicitly was studied later that year by Shorthouse *et al.* [57]. At this point, alpha, beta and delta waves had arisen and expanded rapidly, allowing us to validate the features that defined a variant of concern and how to include this information in the assessment of upcoming variants of interest. This study performed saturation mutagenesis with analysis of folding energy in the then current variants of concern/interest, and the mutational states that represented transitions between different forms. This work was able to use known successful variants to show that strongly destablizing mutations were excluded from the existing dominant variants, whilst stabilizing mutations were enriched. Intriguingly, grouping by spatial location showed that destabilizing mutations were clustered around regions known to interact with host proteins, suggesting that they were tolerated due to compensatory mechanisms that counterbalance any loss of fitness. All three studies inspired an in depth methodological analysis, spurred by the availability of multiple structures of COVID spike [58]. Assessing multiple structures leads to variation in energy calculations, and this is a potential source for bias or error in each study. Whilst the work confirmed the analyses presented, it highlighted the sensitivity of the approach to structural availability and potential for bias. This demonstrates a challenge for folding calculation methods, particularly where structures are derived at a range of qualities, with different co-factors and experimental techniques, and should be a target for future study. As with other studies this work supported sequencing efforts in new samples [59], and the open question left about binding affinities was addressed by later studies [60]. Finally, the work was expanded upon by explicitly considering the role of glycosylation [61], where once again stability was found to be a key factor.

## MISFOLDING WITHIN LARGE SETS OF GENES

As illustrated above, computational saturation screens of single genes offers a powerful tool for understanding the link between function and phenotype. It further opens the opportunity for studies that aggregate data to identify broader trends or distinct properties. One of the earliest examples of this was a ‘structural systems biology’ approach taken by Cheng *et al.*, which studied the MAPK pathways and the yeast cell cycle with a combined misfolding screen and ordinary differential equation network modelling approach [62]. ΔΔG values were combined with a measure of systemic control to determine a ‘systemic impact factor’ (SIF), used to introduce structural effects into the network model. In yeast they were able to show that these SIF values correlated to experimental measurement of *in vitro* cell length for models of the cell cycle at a restrictive temperature. Studies of MAPK signalling revealed distinct effects of *HRAS* mutations from other pathway members.

Kiel *et al.* took a comparable approach to comparing mutations of the RAS pathway in cancer and the RASopathies [11]. The RAS pathway is heavily mutated in a wide range of cancers, whilst RASopathies are a diverse set of common and rare germline conditions arising from single mutations to RAS pathway members. Curiously, whilst RASopathies are associated with an increased risk of cancer (alongside other clinical phenotypes), the overlap between cancer associated mutations and RASopathies is weak and raises the question of whether it reflects some fundamental difference between the mutations. This study showed that cancer causing mutations had a greater misfolding energy, and a network modelling approach suggested that the mutations associated with RASopathies caused relatively minor pathway dysregulation. They further found that experimentally determined rate constants correlated with misfolding energies determined by FoldX.

Gerasimavicius *et al.* took this approach further to study the mutational landscape of all pathogenic mutations over two studies. In the first multiple protein stability predictors were competed to assess their ability to predict pathogenicity and secondly to explore the patterns of mutations that determine loss versus gain of function [63, 64]. The second study highlighted distinct distributions and patterns of pathogenic mutations, with separate clustering of different classes of pathogenic mutations in space and with different degrees of structural perturbation. Separating the pathogenic mutations further into recessive versus dominant classes they found mutation class specific features, observing that dominant negative mutations cluster at the interface between subunits in oligomeric complexes, whilst autosomal recessive mutations tend to reside within the protein interior. Notably, gain of function mutations made more subtle alterations to the protein structure, consistent with the observations made by Kiel in RASopathies [11].

Finally Hall *et al.* presented an analysis and software tool for analysis of mutations observed in aged tissues, effectively using *in vivo* fitness as a genetic screen [65], based on prior work studying such systems [66]. Aged tissues are a mosaic of clones carrying mutations, where only those with a selective advantage are able to persist and expand over time. Missense mutations are common, and are observed to occur in known biologically important regions of mutated proteins. The statistical approach taken in this work revealed an apparently worrying contradiction—mutations to *NOTCH1* at known important interfaces were unexpectedly not statistically enriched relative to a null hypothesis. This could be understood however through the use of folding calculations—misfolding as a mechanism for causing loss of gene activity was so dominant that it acted as a confounder. Once mutations that induced misfolding were excluded from calculations, the relative enrichment of different molecular mechanisms, including JAGGED and calcium binding sites, was revealed. This study further observed a trend for increased misfolding in fitness supressing and fitness enhancing mutations, validated in mutations found in a mouse model of ageing, comparable to the patterns of loss of function versus gain of function mutations observed in Gerasimavivius *et al.* [63].

## FUTURE DEVELOPMENTS

As a tool for understanding mutagenesis, misfolding calculations are now well established. As they are used increasingly in exhaustive screens, it is an open question how the technology will develop and continue to be applied in future. One key feature that reflects its maturity is the range of tooling that is becoming available, particularly evident in the analyses of COVID spike protein, as scripts developed by different groups become refined. MutateX [67] is one example of a devoted set of scripts to automate the calculation of saturation screens, validated against several well understood systems. Related to folding calculations, the same tools can further be adapted to calculations of binding affinity, as achieved in RosettaDDG, a Rosetta based pipeline for calculating folding energies and affinity [17]. As high-quality models of protein structure have become more widely available due to alphafold predictions [68, 69], we can further expect more studies that perform calculations on these models, as has been done with neurexin [70]. An additional likely future development is the incorporation of these calculations into existing machine learning methods for variant prediction. As folding energy has been demonstrated to be descriptive for predicting mutation effects, it follows that this energy would be a useful additional feature for these tools. For many frequently mutated genes, there would also be a value in pre-calculating folding energies and making them available through public databases.

**Figure 2:**
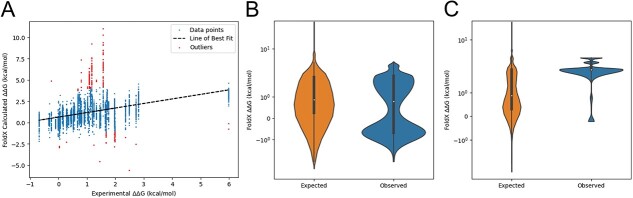
Exploring challenges for prediction in future. **(A)** Estimates of correlation between experimental and calculated values of ΔΔG of mutation can be low for individual proteins, but this may not substantially limit their ability to classify. A systematic analysis of ΔΔG analyses applied to structures of PIN1 shows a low Spearman correlation of 0.46. However, a linear regression highlights that 80% (4/5) outliers (red) result from four substitutions of ~800. **(B)** Gain of function mutations may not show the same enrichment for misfolding as loss of function mutations. PTPN11 ΔΔG estimates here show a distinct distribution from the null hypothesis when applied to the whole gene, with a shift towards stabilization. **(C)** PTPN11 ΔΔG estimates when applied to the autoinhibitory SH2 domain show that misfolding is favoured.

Substantial challenges and questions still remain however for the method. One noteworthy feature is that whilst there have been successes in developing folding based classifiers, correlations reported in the literature between theoretical predictions and experimental observations vary widely, for example, 0.2, 0.5 and 0.8 depending on structure [24, 71, 72]. This would suggest that success depends at least in part on the gene of interest and whether its structure is amenable to computational saturation screens (e.g. globular proteins are likely to perform better). We note that correlation, however, may not be an appropriate tool in isolation to measure success, as our comparison of theoretical and experimental estimates from different structures of PIN1 reveals that 80% (4/5) of outlier estimates arise from only 4 of ~800 substitutions (Figure 2A). Further systematic analysis of multiple structures, such as [58, 73], would support our understanding of the limitations of FoldX and other predictive tools.

A further issue is how applicable this approach will be for the analysis of pathogenic gain of function mutations. It is intuitive that misfolding can cause loss of gene activity, but there is some evidence that alterations of folding of specific domains may enable gain of function. This was explored in the selection of mutations to *FBXW7* in aged skin [65], which may be expected to have gain of function mutations under positive selection due to its relationship with *NOTCH1* in other tissues. In this situation, apparent selection of stablizing mutations across the gene was influenced by the strong selection of non-destabilizing mutations to the substrate binding site, and was not apparent once they were excluded. More generally, we might expect that for gain of function mutations, the role of specific domains or regions in gene function becomes more important (as suggested in [63]). For example, mutations to *PTPN11* can lead to different RASopathies through gain of function. Whilst the impact of mutation on misfolding across the whole protein sequence shows a selection for stablization of the protein (Figure 2B), mutation to the autoinhibitory SH2 domains shows strong selection of destabilizing mutations (Figure 2C). Dedicated studies of well characterized gain of function mutations in single genes will better illuminate the utility of folding calculations here.

A more complicated question is what the role of these folding calculations is in light of more advanced experimental techniques for measuring folding. Tsuboyama *et al.* recently published a landmark paper presenting cDNA display proteolysis, a technique for rapidly measuring folding stability [74] applied to the study of single and double mutants. This work further presents a uniquely powerful resource for understanding protein stability for a large set of proteins measured under consistent conditions. In the context of this and other folding based deep mutation scans, the question arises—do we need to attempt to predict what we can measure? Just as the widespread availability of high-quality protein structure models from Alphafold [68] does not negate the value of novel experimentally derived structures, experimental tools to measure protein folding effects of mutations do not negate the utility of computational predictions of folding energy. Theoretical approaches offer unique insights into molecular mechanisms alongside experimental data, and both schools have a long tradition of being accelerated by the availability of new technologies, through working synergistically together. Two further factors also challenge the primacy of single experimental approaches. Experimental assays have inherent limitations, which have been noted to reflect lab specific experimental conditions [25], and even highly similar but different tissue environments influence patterns of mutagenesis *in vivo* [65]. As such it cannot be assumed that single assays are ‘correct’ for all situations. Second, imperfect measures (whether experimentally or computationally determined) have been shown to be highly effective, as illustrated by examples cited here that use homology models successfully in the absence of experimental structures [25]. One clear target for combined computational and experimental targets to address is the origin of the apparent gap between statistically derived pathogenicity scores, particularly as recent methods show increasing accuracy [75]. In the longer term, we can expect folding forcefields and calculation methods to improve, and folding and dynamics calculations will continue to offer a uniquely powerful and relevant window into experimentally inaccessible problems.

**Key Points**

- The estimation of folding energies through general forcefields has become increasingly accurate whilst computing costs have continued to come down.
- This enables the application of computational saturation screens that explore the mutational landscape of different genes.
- For individual genes, this has enabled the creation of predictive screens that support analysis of newly observed variants.
- It further enables powerful aggregate analysis of collections of mutations across multiple genes, revealing fundamental shared features and interactions across networks.

## Author Biographies

**David Shorthouse** is a lecturer at UCL using data science to study cancer patient heterogeneity.

**Harris Lister** and **Gemma Freeman** are undergraduate students currently studying Biomedical Engineering and Infection and Immunity at UCL, respectively. Ms Freeman is a Laidlaw Scholar.

**Benjamin A. Hall** is a principal research fellow at UCL, using computational models to study how mutations alter cells in healthy ageing and carcinogenesis.
